# Comprehensive Profiling of Microbiologically Induced CaCO_3_ Precipitation by Ureolytic *Bacillus* Isolates from Alkaline Soils

**DOI:** 10.3390/microorganisms9081691

**Published:** 2021-08-09

**Authors:** Olja Šovljanski, Lato Pezo, Jovana Stanojev, Branimir Bajac, Sabina Kovač, Elvira Tóth, Ivan Ristić, Ana Tomić, Aleksandra Ranitović, Dragoljub Cvetković, Siniša Markov

**Affiliations:** 1 Faculty of Technology Novi Sad, University of Novi Sad, Bulevar Cara Lazara 1, 21000 Novi Sad, Serbia; ivan.ristic@uns.ac.rs (I.R.); anav@uns.ac.rs (A.T.); a.ranitovic@uns.ac.rs (A.R.); cveled@uns.ac.rs (D.C.); sinisam@uns.ac.rs (S.M.); 2 Institute of General and Physical Chemistry, Studenski Trg 12/V, 11000 Belgrade, Serbia; latopezo@yahoo.co.uk; 3 BioSense Institute, University of Novi Sad, Dr Zorana Đinđića 1, 21000 Novi Sad, Serbia; jovana.stanojev@biosense.rs (J.S.); branimir.bajac@biosense.rs (B.B.); 4 Department of Crystallography and Mineralogy, Faculty of Mining and Geology, University of Belgrade, Đušina 7, 11000 Belgrade, Serbia; sabina.kovac@rgf.bg.ac.rs; 5 Department of Physics, Faculty of Science, University of Novi Sad, Trg Dositeja Obradovića 3, 21000 Novi Sad, Serbia; elvira.djurdjic@df.uns.ac.rs

**Keywords:** MICP process, kinetic study, calcite, vaterite, MALDI-TOF identification, *Bacillus licheniformis*, *Sporosarcina pasteurii*

## Abstract

Microbiologically induced CaCO_3_ precipitation (MICP) is a well-known bio-based solution with application in environmental, geotechnical, and civil engineering. The significance of the MICP has increased explorations of process efficiency and specificity via natural bacterial isolates. In this study, comprehensive profiling of five soil ureolytic *Bacillus* strains was performed through a newly formed procedure that involved six steps from selection and identification, through kinetic study, to the characterization of the obtained precipitates, for the first time. To shorten the whole selection procedure of 43 bioagents with the MICP potential, Standard Score Analysis was performed and five selected bacteria were identified as *Bacillus muralis*, *B. lentus*, *B. simplex*, *B. firmus*, and *B. licheniformis* by the MALDI-TOF mass spectrometry. Despite following the targeted activity, kinetic studies were included important aspects of ureolysis and the MICP such as cell concentration, pH profiling, and reduction in calcium ion concentration. At the final step, characterization of the obtained precipitates was performed using FTIR, XRD, Raman, DTA/TGA, and SEM analysis. Although all tested strains showed significant potential in terms of precipitation of calcite or calcite and vaterite phase, the main differences in the MICP behavior can be observed at the bacterial strain level. *B. licheniformis* showed favorable behavior compared to the reference *Sporosarcina pasteurii* DSM 33.

## 1. Introduction

The presence of bacteria with biomineralization capability in soils, freshwaters found in lakes, reservoirs, ponds, rivers, streams, wetlands, and even groundwater and saline habitats has been related to the formation of different minerals around living cells under varied environmental conditions. Due to this fact, these bacteria represent resourceful bioagents for engineering approaches, and consequently, microbiologically induced carbonate precipitation (MICP) has been extensively studied at different levels of application in environmental, geotechnical as well as civil engineering [1,2]. Through the MICP process, bacteria can modify soil characteristics resulting in stabilization from the action of wind and water erosion [3], removing heavy metals from wastewaters [4], or promoting the self-healing effect of calcareous materials [5]. The water absorption of the monumental limestone was reduced by 60% after the MICP treatment by *Micrococcus* sp. isolated from a historical site [6]. The high rate of self-healing effect of mortar was achieved using granulated denitrifying soil microbial culture [7], while Achal et al. [8] performed a phenotype modification of *Sporosarcina pasteurii* with UV radiation, which resulted in the alkaline resistant mutant at pH values above 11, with pronounced ureolytic activity and biocalcification in sand consolidation treatment.

The wide range of possibilities in applications of the mentioned bacteria type is based on metabolic activities involved in the MICP process. Whether the metabolic pathway is autotrophic (induction of local CO_2_ reduction) or heterotrophic (lead to active and passive CaCO_3_ formation), the MICP process is a consequence of carbonate production by ion transport across cellular membrane and reactivity of calcium ions accumulation on the surface of negatively charged bacterial walls [9]. Furthermore, the whole process is reflected in a change of pH value and concentration of dissolved inorganic deposits in a matrix, which is especially expressed during ureolytic activity [2]. Therefore, kinetic pathways of ureolytic activity have been used to better understanding the MICP process based on predictions, optimization, and description of the kinetic parameters [10]. Although the kinetic study can vary widely between bacterial species [3], the activity of high-efficiency urease producers has been observed as the Michaelis-Menten model and described by the highest enzyme reaction rate (v_max_) and the half-saturation coefficient (K_m_) [11]. When it comes to the use of bacteria in engineered MICP processes, the selection of bacteria is based on some beneficial traits that make them suitable for the application include adaptability to different ecological conditions, efficiency metabolic activity, short generation time, sporulation ability, etc. [12]. Therefore, isolation of these bacteria was performed from areas rich in carbonate rocks such as mines [4], soils [13], landfills [14], cement matrix [15], sand [16], active sludge from wastewater treatment [17], etc.

However, most research studies are focused on MICP applications as well as the determination of mechanisms for process control. Very few studies are oriented to comprehensive profiling of the MICP through the formation of the whole procedure for the selection of the most potent bacteria for different purposes. In this study, 43 bacterial isolates were investigated through six experimental steps which involved a statistical approach to the selection, bacterial identification, and examination of metabolic activities and kinetic pathways relevant to targeted processes. Furthermore, the complete characterization of the obtained precipitates was performed using X-ray diffraction (XRD), Fourier transform infrared spectroscopy (FTIR), Raman analysis, differential thermal and thermogravimetric analysis (DTA/TGA), and scanning electron microscopy (SEM).

## 2. Materials and Methods

**Figure 1:**
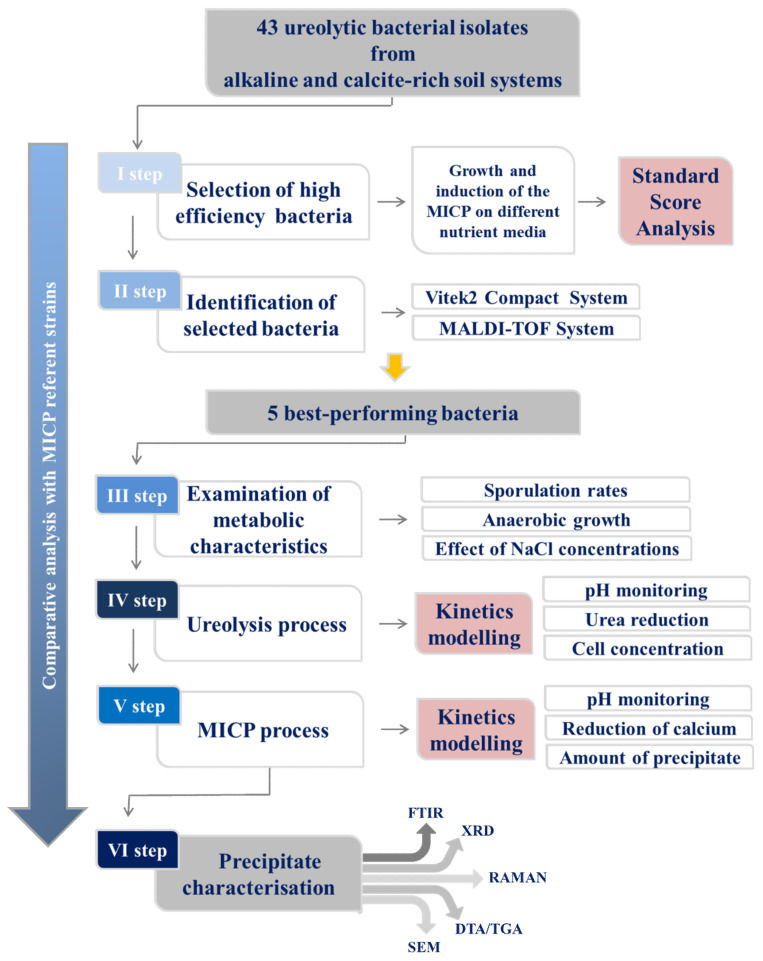
Experimental setup.

A total number of 43 bacterial isolates (I_1_-I_3_; II_1_-II_10_; III_1_-III_20_; IV_1_-IV_10_) from soil samples were analyzed and characterized as alkaline-adapted and sporogenic bacteria with growth possibility over a wide range of temperatures and pH values [18]. *Sporosarcina pasteurii* DSM 33 was used as a positive control for the MICP through ureolysis, while carbonate-induced bacterium *Bacillus pseudofirmus* DSM 8715 was a negative control for ureolytic activity. The mentioned groups of bacterial isolates were well as referent controls were the subject of this study, while the experimental setup is schematically shown in Figure 1.

### 2.1. The First Step: Selection of the Bacterial Isolates 

In order to shorten the procedure for the selection of the bacteria with biomineralization capacity, different metabolic activities were examined through Standard Score Analysis. The standard scores were calculated for the evaluation of the MICP efficiency. The samples were normalized, using extreme values of the MICP assays, while the dimensionless scores were evaluated by subtracting the minimum values from the raw data means and dividing it by the obtained range for each assay [19], according to Equation (1).

(1) $${\bar{x}}_{i}=\frac{x_{i}-\underset{i}{\min}x_{i}}{\underset{i}{\max}x_{i}-\underset{i}{\min}x_{i}},\;\forall i$$

The standard score (SS) is the mathematical function whose maximum has been determined by evaluating the scores for all variables, according to Equation (2). Each output variable has its weight ($w_{i}$) when calculating the function SS:

(2) $$\begin{array}{l} \mathrm{SS}=w_{1}\cdot \bar{\mathrm{SR}}+w_{2}\cdot \bar{\mathrm{pH}}+w_{3}\cdot \bar{\mathrm{UAG}}+w_{4}\cdot \bar{\mathrm{UAC}}+w_{5}\cdot \bar{\mathrm{UBT}}\\ \;+w_{6}\cdot \bar{\mathrm{UBC}}+w_{7}\cdot \bar{\mathrm{UBCA}}+w_{8}\cdot \bar{\mathrm{MUC}}+w_{9}\cdot \bar{\mathrm{MEC}} \end{array}$$

where: sporulation rate ($\bar{\mathrm{SR}}$), optimal pH growth value ($\bar{\mathrm{pH}}$), growth on Urea agar ($\bar{\mathrm{UAG}}$), Urea agar color change ($\bar{\mathrm{UAC}}$), Urea broth turbidity ($\bar{\mathrm{UBT}}$), Urea broth color change ($\bar{\mathrm{UBC}}$), addition of CaCl_2_ in inoculated Urea broth ($\bar{\mathrm{UBCA}}$), CaCO_3_ formation on/in Urea-CaCl_2_ medium ($\bar{\mathrm{MUC}}$), and CaCO_3_ formation on/in YE-CaCl_2_ medium ($\bar{\mathrm{MEC}}$) present the normalized values of experimentally obtained variables, obtained using Equation (1). The maximum of SS function presented the optimal parameters and also the optimum for parameters. If the value of optimal function was close to 1, it showed the tendency of tested MICP parameters of being optimal. 

For examination of the MICP potential, bacteria were grown on: (i) Urea base agar and broth (HiMedia, Mumbai, India), (ii) Urea-CaCl_2_ agar and broth [20], and (iii) YE-CaCl_2_ agar and broth (modified of Urea-CaCl_2_ media; Urea was replaced with 4.0 g/L yeast extract). Overnight bacterial suspension aliquots (0.3 mL) were transferred into previously mentioned liquid media, adequate dilutions were made, and enumeration of bacteria was performed by streaking on TSA with the addition of 20% Urea. After an incubation period of 7 days at 30 °C, the turbidity, color change (equivalent to pH changes), and addition of 3.2 g/L CaCl_2_ (rapid test for precipitation) were observed in the Urea base broth. The precipitation of inorganic deposits was observed in Urea-CaCl_2_ and YE-CaCl_2_ broth. A total volume of inoculated media was centrifuged (10,000 rpm, 15 min) and the deposits were washed out three times by sterile distilled water, dried to constant mass. The precipitates were subjected to FTIR spectrophotometric analysis (Hartmann & Braun MB-series). The bacterial growth on Urea base agar, as well as colour changes, were checked after 7 days at 30 °C, while inorganic depositing on solid media with calcium ions was observed by light microscope (Zeiss Primo Star Binocular Microscope, Jena, Germany). Based on standard score results, bacterial isolates with the best performance based on tested characteristics will be chosen for the next experimental steps.

### 2.2. The Second Step: Bacterial Strain Identification

Primary identification by VITEK^®^ 2 Compact System (BioMérieux, Carpone, France) was performed following standard procedure using VITEK^®^ 2 BCL ID cards (BioMérieux, Carpone, France). Additionally, identification by MALDI-TOF mass spectrometry was performed following Bruker’s direct transfer sample preparation procedure. MALDI-TOF mass spectra were obtained by using MicroflexLT/SHBioTyper spectrometer (BrukerDaltonics, Billerica, MA, USA) with a nitrogen laser (337 nm). Spectra acquisition in the mass range 2–20 kDa was collected using the Auto Execute option by accumulating 240 laser shots (laser frequency, 60 Hz; ion source 1 voltage, 19.9 kV; ion source 2 voltage, 18.53 kV; lens voltage, 6 kV) acquired at 30%–40% of maximum laser power. VITEK Compact identification provides information of identification probability (>90% are high-confidence), while MALDI-TOF identification provides score value (0–1.69 no identification possible; 1.70–1.99 low-confidence identification; 2.00–3.00 high-confidence identification). For both identification methods, the bacterial cultures were grown overnight on Columbia agar +5% sheep blood (BioMérieux, Carpone, France) at 30 °C.

### 2.3. The Third Step: Examination of Metabolic Activities Relevant to the MICP

The effect of different NaCl concentrations (3%, 5%, 7%, 10%, and 15%) on bacterial viability was observed as turbidity of inoculated TSB (Tryptone Soya Broth, HiMedia, Mumbai, India) after 48 h at 30 °C. The Anaerocult A (Merck, Kenilworth, NJ, USA) was used for checking the possibility of bacterial growth in anaerobic conditions. The bacterial cultures were streaked onto TSA with an addition of 20% Urea and incubated for 5 days at 30 °C in the anaerobic jar. Sporulation rates for selected strains as well as reference cultures were examined at 30 °C on two different pH values (7.3 and 9.3) in TSB with an addition of 20% Urea. The increasing pH value of medium to 9.3 was performed with the addition of 1 M sodium sesquicarbonate. The samples were taken periodically during 7–days incubation at 30 °C, subjected to thermal treatment to select only sporogenic forms, and streaked onto TSA with the addition of 20% Urea. During thermal treatment (80 °C, 10 min) of bacterial suspension of sporogenic and vegetative cells, only spores can survive in this way.

### 2.4. The Fourth Step: Kinetics of Ureolysis and pH Monitoring

The ureolysis rates were determined by Urea/Ammonia (Rapid) Assay Kit (Megazyme, Vienna, Austria) following the assay procedure. Urea broth was inoculated with 10% *v*/*v* freshly prepared bacterial suspension (approx. 6 log/mL) and Urea concentrations were followed during 72 h at 30 °C (0, 1, 4, 6, 8, 10, 12, 24, 36, 48, 60, and 72 h). Every aliquot was sterile filtered and optionally diluted to determine the absorbance at 340 nm. The pH value was measured at the same sampling time using pH-meter HI99161 (Hanna Instruments, Woonsocket, RI, USA). The number of viable cells was determined by the streaking method into triplicate on TSA with Urea and incubated 24 h at 30 °C.

The evaluation of the rate coefficient for ureolysis was performed by integrating the first-order ordinary differential Equation (3) in which the constant number of viable cells during the period of Urea hydrolysis was assumed [21].

(3) $$\frac{dc_{Urea}(t)}{dt}=-k_{Urea}\cdot c_{Urea}(t)\cdot c_{X}\;\Rightarrow c_{Urea}(t)=c_{Urea}(0)\cdot \exp\left(-k_{Urea}\cdot t\cdot c_{X}\right)$$

The coefficient *k_Urea_* is the first-order rate coefficient for ureolysis, while *t* and *c_X_* are time and the numbers of viable cells, respectively. A first-order relationship has been successfully used to describe bacterial ureolysis [22,23]. The kinetics of pH values and numbers of viable cells during the process was investigated according to the four-parameter sigmoidal mathematical model Equation (4) recommended by Romano et al. [24] since is suitable for biologic systems.

(4) $$y(t)=d+\frac{a-d}{1+{\left(\frac{t}{c}\right)}^{b}}$$

In Equation (4), *y*(*t*) presents pH values or numbers of viable cells, a and d represent the minimum and maximum value that was obtained, respectively, c refers to the inflection point (the point on the S-shaped curve between a and d, while b is the Hill’s slope (slope factor) of the curve (the steepness of the curve at point c).

### 2.5. The Fifth Step: Evaluation of the MICP Efficiency

#### 2.5.1. Precipitation Rate Kinetics

For the MICP quantitative test, all tested bacterial strains were incubated in Urea-Ca^2+^ broth at 30 °C for 14 days. The amount of inorganic deposit, pH value, and concentration of free calcium ions were determined at the beginning of the incubation and periodically from the first to the fourteenth day of incubation. At each sample time, the pH values were measured, and the total volume of inoculated broth (100 mL) was centrifuged (10,000 rpm, 15 min). Any mineral deposits adhered to the bottle walls were removed by scraping with a sterile inoculated needle. The pellets were centrifuged to remove biomass and extra nutrients, washed with sterile distilled water, and dried to constant mass. The amount of inorganic deposit and the kinetics of pH value were investigated similarly to Section 2.4, where the four-parameter sigmoidal mathematical model was used.

The CaCO_3_ precipitation is dependent on the saturation state, as well as the CaCO_3_ growth mechanism [23]. A non-affinity-based first-order rate law could be applied to describe the data adequately. Briefly, an assumption was made that 1 mol CaCO_3_ was formed after each mole of CaC_2_ was removed from the solution [25]. After the integration, Equation (5) is used, where *k_precipitate_* is the first-order rate coefficient for CaCO_3_ precipitation.

(5) $$\frac{dc_{Ca^{2+}}(t)}{dt}=-k_{precipitate}\cdot c_{Ca^{2+}}(t)\;\Rightarrow c_{Ca^{2+}}(t)=c_{Ca^{2+}}(0)\cdot \exp\left(-k_{precipitate}\cdot t\right)$$

Rate constants for the evaluation of ureolysis and reduction in calcium ions were presented using a nonlinear regression method using the Solver function in Microsoft Excel 2019.

#### 2.5.2. Determination of Calcium Concentration

The sterile filtration (filter pore 0.22 µm) of supernatant was performed and the concentration of free calcium ions was analyzed by atomic absorption spectrophotometry (Spectra 220FS, Varian, Palo Alto, USA) with direct suction.

### 2.6. The Sixth Step: Characterization of Precipitates

To characterize and compare the precipitates obtained in Section 2.5.1. during the incubation period, the samples were tested after the first and the last day of the incubation period. To analyze the mineralogy of the samples, X-ray powder diffraction (XRD) and Fourier transform infrared spectroscopy (FTIR) techniques were performed using an XRD diffractometer (Rigaku SmartLab with CuKα radiation) and Bomem FTIR spectrophotometer (Hartmann & Braun MB-series). All XRD results are processed using Rigaku PDXL 2: Integrated powder X-ray diffraction software, Version 2.8.3.0. (Rigaku Corporation, Tokyo, Japan) as well as card standards (calcite: 01–072–1652, vaterite: 01–075–9355, and vaterite: 01–075–9356). The chemical bond vibrations during FTIR analysis were recorded in the range of 4000–400 cm^–1^, using the ATR technique. Additionally, Raman analysis was performed at a range of 100–1500 cm^–1^ using a DXR Raman microscope (Thermo Scientific, Waltham, MA, USA). The thermal properties were investigated using differential thermal and thermogravimetric analysis (DTA/TGA) which was performed in the temperature range up to 1000 °C with the heating rate of 20 °C/min in a nitrogen atmosphere (30 mL/min) using TGA-DSC/DTA coupled system—NETZSCH STA 449 F5 Jupiter combined with DSC 204 F1 Phoenix. The morphology was analyzed using scanning electron microscopy (SEM Hitachi TM3030) under a high vacuum (acceleration voltage 15 kV, beam current 20 nA, spot size 1 mm) and a LeyboldHeraus L560Q sputter coating device was used for imaging and covering samples with gold, respectively.

## 3. Results and Discussion

### 3.1. Selection of the Bacterial Isolates

A total of 43 alkalophilic, sporogenic bacteria were subjected to physiological and metabolic analyses on different nutrient media and compared to ureolytic *S. pasteurii* DSM 33 and non-ureolytic reference *B. pseudofirmus* DSM 8715. In the past two decades, both referent bacteria are indicated as an effective MICP bioagent through ureolysis or organic acid oxidation pathway [12,26,27,28,29].

**Figure 2:**
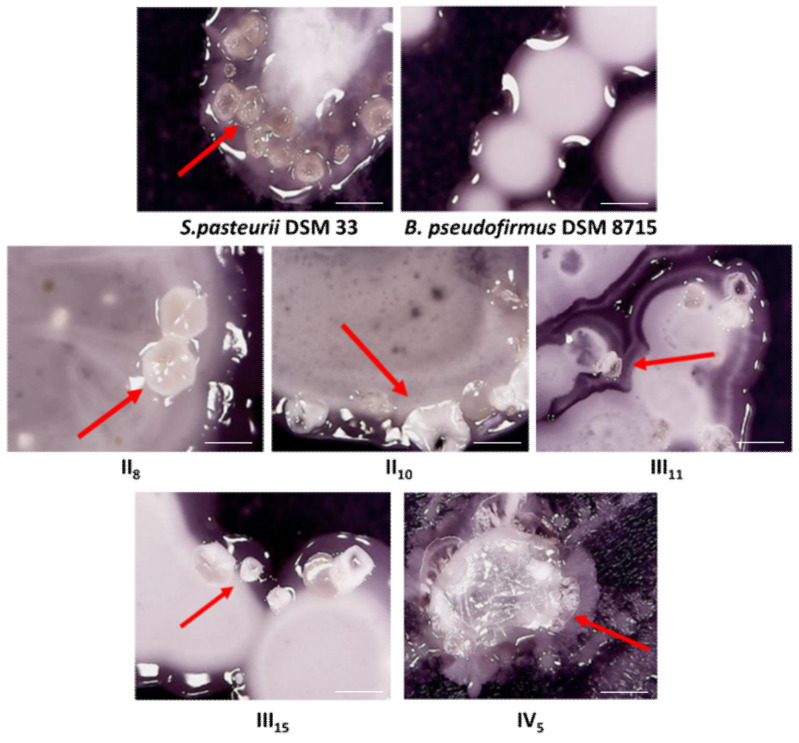
Optical microscopy of bacterial colonies on Urea-CaCl_2_ agar (400 × magnifications; scale bar 2 mm; red arrow indicated crystal formation with different positions relative to the colony center).

In the initial step of this study, the assessment of bacteria based on biomineralization capacity was a priority. Using the obtained results of growth and behavior on different nutrient media for biomineralization as well as alkaline and sporogenic characteristics, the Standard Score Analysis of the MICP variables was accomplished. The optimal ranges for the output parameters are presented in Supplementary Table S1, while the maximum of experimentally obtained values was considered optimal (all polarity values were positive). The minimum and maximum of all observed variables obtained during the experiment are shown in Supplementary Table S1. Based on Standard Score Analysis, five bacterial strains were selected as potentially a suitable choice for the MICP process. The selected strains had a standard score (rang of samples according to the deviation and the mean) of 0.996, while a standard score for *S. pasteurii* DSM 33 and *B. pseudofirmus* DSM 8715 are 0.775 and 0.726, respectively. The greater standard score for bacterial isolates was reached because these five bacteria were effective in inducing crystal formation on nutrient media with and without Urea, while referent strains induced precipitation on only one type of media. This suggests that referent bacteria have the opportunity to induce CaCO_3_ precipitation only during ureolysis or oxidation of organic acid, while the best-performing bacterial isolates can use both metabolic pathways for the targeted process. Except for statistical similarity, related behavior of selected strains and ureolytic reference was observed onto the solid nutrient medium rich in Urea (Figure 2). The crystal forms (marked with red arrows in Figure 2) were observed as reflected solid structures of each colony, but with different positions relative to the colony center. Furthermore, the shape and size of crystal forms were different on the bacterial strain level. As expected, the inorganic deposit was not observed only in the case of *B. pseudofirmus* DSM 8715.

### 3.2. Bacterial Strain Identification

The five bacterial isolates (previously named II_8_, II_10_, III_11_, III_15_, and IV_5_) have been selected for the identification process. According to the gained results, the identification by VITEK2 Compact System was not precisely identified all isolates. In summary, the only one isolate II_8_ was identified as *Brevibacillus chosinensis* with a 91% probability. For the rest of the isolates, the VITEK identification was presented with at least two options (bacterial strain II_10_ was identified as *Bacillus fordii* or *Geobacillus toebii*, stains III_11_ and III_15_ as *B. badius*, *B. simplex,* or *B. chosinensis,* while strain IV_5_ was recognized as *Lysinibacillus sphaericus* or *L. fusiformis*). Contrarily, the MALDI-TOF analyses were provided complete identification with a high score value for the best match. All tested bacteria belong to genera *Bacillus*, which were previously reported as spore-formatting species with a high surviving level in alkaline environments and with the capability to do the MICP effectively [30]. The bacterial strains II_8_ and II_10_ are identified as *B. muralis* and *B. lentus* (identification scores were 2.76 and 2.28, respectively), while isolates III_11_, III_15_, and IV_5_ are *B. simplex*, *B. firmus*, and *B. licheniformis*, with identification scores of 2.04, 2.81, and 2.43, respectively. These *Bacillus* strains were previously reported in scientific literature and described characteristics are in correlation with the obtained in this study. Briefly, *B. muralis* isolated from historical building material [31], as well as soil isolates *B. muralis* and *B. licheniformis* showed high potential in inducing precipitation of calcium carbonate [32]. Vahabi et al. [33] reported the isolation of *B. licheniformis* from limestone cave soil and its application in the MICP, while Enyedi et al. [34] emphasized the role of *Bacillus simplex* isolated from cave deposits in the MICP process. Ureolytic strain *B. lentus* from marine sediment demonstrated great potential in CaCO_3_ precipitation [35], while cave isolate *B. firmus* was able to mediate precipitation during in vitro experiments [36].

According to Timperio et al. [37], MALDI-TOF analysis showed a high similarity with 16S rDNA analyses, suggesting the applicability for low-cost and rapid screening of bacteria from extreme environments. Since the MALDI-TOF identification is well-matched with a large range of culture media and conditions and also has a customized base for a large number of bacteria from different sources [37], the obtained disagreements between results of applied identification methods were expected. Furthermore, Guo et al. [38] labelled that the MALDI-TOF system offers higher identification accuracy and lower error rates at the species level of wild isolates compared to VITEK2 Compact System. It might be concluded that MALDI-TOF identification has a better quality of base for the identification of non-clinical isolates than the VITEK2 Compact System. For this reason, but also since the obtained identification by the MALDI-TOF system had mostly high identification scores, MALDI-TOF results were adopted as adequate species-level identification. Using the MALDI-TOF identification, Bibi et al. [3] were also identified ureolytic bacteria with the MICP capability as *B. cereus*, *B. licheniformis*, and *B. subtilis*, while Abdel Samad et al. [39] reported the identification of *Virgibacillus* sp., *B. circulans*, *B. licheniformis*, and *B. cereus* as mineral-forming bacterial isolates from sabkhas.

### 3.3. Examination of Metabolic Activities Relevant to the MICP Process

Different physiological and ecological tests might be important for understanding behavior during the MICP process [40]. Due to this hypothesis, bacterial growth in oxygen-free conditions, salts tolerance, and sporulation rate is observed as important factors affecting the efficiency of precipitation around bacterial cells. The bacterial growth in the absence of oxygen did not appear after 5 days incubation period which might limit the potential use of these bacterial strains in the subsurface and oxygen-free processes. According to Nakbanpote et al. [41], salt tolerance can be correlated with a bacterial ability to form biofilms, which is important for plant growth-promoting bacteria and the possibility to survive salinity stress in a soil environment. Moreover, due to salt-affected land areas in the world, many Gram-positive and Gram-negative bacteria isolated from agricultural soils have been able to adapt up to 10% of NaCl in soils [41,42]. According to the gained results of salt tolerance, *B. muralis* and *B. simplex* were able to grow in presence up to 10% NaCl, while *B. lentus* and *B. licheniformis* can tolerant up to 7% NaCl. *B. simplex* was determined as the lowest salt-tolerant isolate (up to 5%). Interestingly, *S. pasteurii* DSM 33 can grow only in present up to 3% NaCl, which is opposite to a high level of salt tolerance (up to 10%) of non-ureolytic *B. pseudofirmus* DSM 8715.

**Figure 3:**
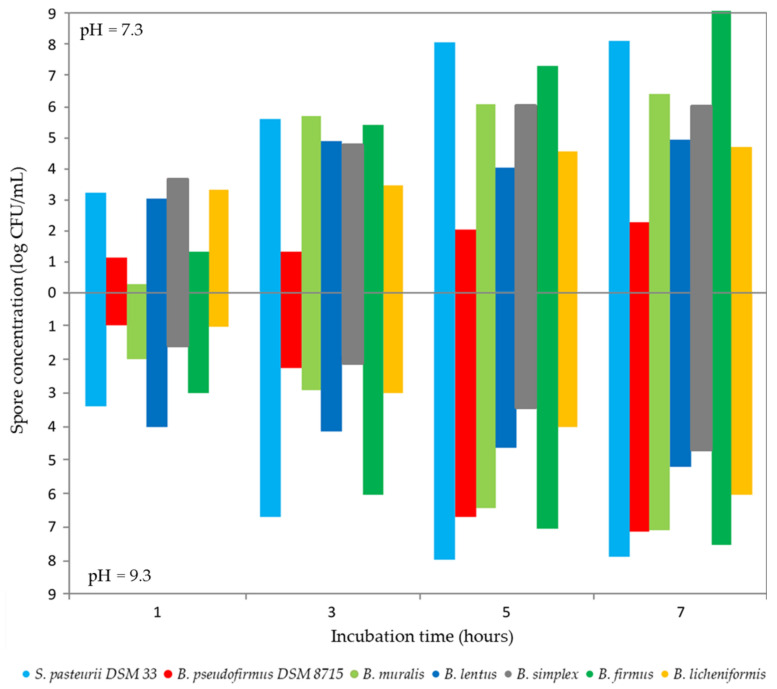
The spore rate in TSB nutrient medium with different pH values.

The spore formation in the MICP engineering application is significant because resistant life forms can survive extreme MICP conditions such as high alkalinity, lower water availability, etc. [43]. In this sense, ensuring sufficient spore concentration should be required for an effective MICP process. Zhang et al. [43] reported that the optimal spore concentration for the MICP is not less than 8 log CFU/mL and can be correlated with the decreasing of the free calcium ions and pH changes. In this study, the sporulation was followed for 7 days at pH values of 7.3 and 9.3 which represent optimal pH values for bacterial growth and ureolytic activity of *S. pasteurii*, respectively [44]. As shown in Figure 3, only *S. pasteurii* DSM 33 and *B. simplex* have reached 8 log CFU/mL at a pH value of 7.3, while other isolates did not cross 6 log CFU/mL after 7 days. After incubation at a pH value of 9.3, only *S. pasteurii* DSM 33 reached the limit of 8 log CFU/mL, while the spore concentrations of other tested bacteria were between 4 and 7 log CFU/mL. The obtained spore concentrations have been achieved by reducing nutrient concentration during long incubation and without the addition of any substance for sporulation inducing (e.g., Mn^2+^ ions), which can be a solution for rapid achievement the sufficient spore concentration in further experiments [45].

### 3.4. Kinetics of Ureolysis and pH Monitoring

**Figure 4:**
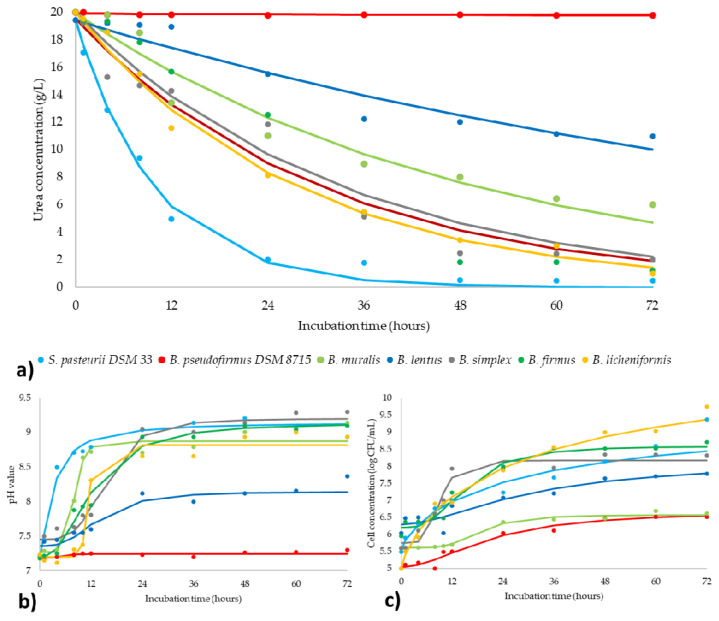
Kinetic study of (**a**) ureolysis process; (**b**) pH value; (**c**) bacteria concentration (markers signify the experimental data; lines indicate predictive results).

Bacteria can promote calcite precipitation by Urea hydrolysis and environment alkalization through the production of NH^4+^ and CO_3_^2−^ ions. Theoretically, it means a direct correlation between the rate of hydrolyzed Urea and the MICP performance [46], while the variance in the rate of pH change and an amount of hydrolyzed Urea between bacterial strains indicates differences in kinetic of ureolytic reaction [23]. In this study, the experimental results of spectrophotometric quantification of Urea concentration were used to define kinetic parameters and fitting the kinetic models. The same process was performed for pH monitoring and concentration of bacteria during the ureolytic activity (Figure 4).

The mentioned parameters are in function of incubation time, including both the experimental data and the plotted curves of kinetic models. Plotted curves for ureolysis (Figure 4a) have a reducing trend because Urea concentration decreased during the whole incubation period. According to the kinetic model present in Figure 4a and Table 1, a statistically calculated Urea concentration fitted in goodly with experimental data. For the proposed kinetic model, all coefficients of determination were greater than 0.883. The regression coefficients of the observed mathematical models were summarized in Table 1 which explains trends (the speed and intensity) of the investigated ureolysis process.

The fit between experimental measurements and model calculated results are given in Supplementary Table S2. The quality of the model fit was tested and the residual analysis of the developed model was also presented in the same table. The presented exponential model (for Urea concentration prediction), and four-parameter sigmoidal mathematical model (for pH value and bacterial concentration prediction) appear to be simple, robust, and accurate. The mathematical models had an insignificant lack of fit tests, which means that all the models represented the data satisfactorily. A high r2 is indicative that the variation was accounted for and that the data fitted satisfactorily to the proposed model.

From the moment of inoculation, the Urea concentration decreased constantly while biomass intensively multiplied for all ureolytic strains (Figure 4). The lag phase in Urea reduction was noticed in the first hour of Urea hydrolysis by *B. muralis*, *B. lentus*, and *B. simplex*, which is a consequence of the existence of the lag growth phase for these bacterial strains. Furthermore, it can also be noticed the moment of progression from the log growth phase to the stationary growth phase for all bacterial strains after 36 h, corresponds to the ending of intensive Urea reduction. The pH changes of the nutrient medium during the incubation period are shown in Figure 4b. The pH value increased gradually with time and reached a constant value within 36 h for all strains. As stated previously, *B. pseudofirmus* DSM 8715 is a non-ureolytic bacterium but has biomineralization capacity [30]. Therefore, this bacterium did not have a response in Urea reduction and pH change, but bacterial viability was detected.

In summary, constant and linear decreasing of Urea concentration was observed for all ureolytic bacteria (Figure 4), while changes in pH value and number of viable cells have followed expected increasing trends. At the end of the incubation period, an amount of Urea residue was between 2.5% and 54.9% of the initial concentration depends on bacterial strain. The greatest similarity in ureolysis was detected between *B. simplex*, *B. firmus*, and *B. licheniformis* (Figure 4a), but with the different final concentrations of Urea: 10.06%, 6.23%, and 5.01%, respectively. Furthermore, these three *Bacillus* strains showed the most similar behavior compared to the reference ureolytic strain *S. pasteurii* DSM 33, and consequently, it can be said that these three strains are the most potent in the view of the ureolysis kinetic pathway.

### 3.5. Evaluation of the MICP Efficiency

In the presence of Urea and calcium ions in a nutrient medium, ureolytic bacteria have the opportunity to produce extracellular carbonate ions and induce precipitation of CaCO_3_ crystals. Moreover, morphology and an amount of precipitate are associated with substrate concentration, environmental parameters as well as inoculation level [47]. For monitoring bacterial behavior and evaluating the biomineralization capacity, the MICP process was followed for 14 days for all selected bacteria as one of the main responses of the MICP efficiency was taken an amount of the precipitate.

**Figure 5:**
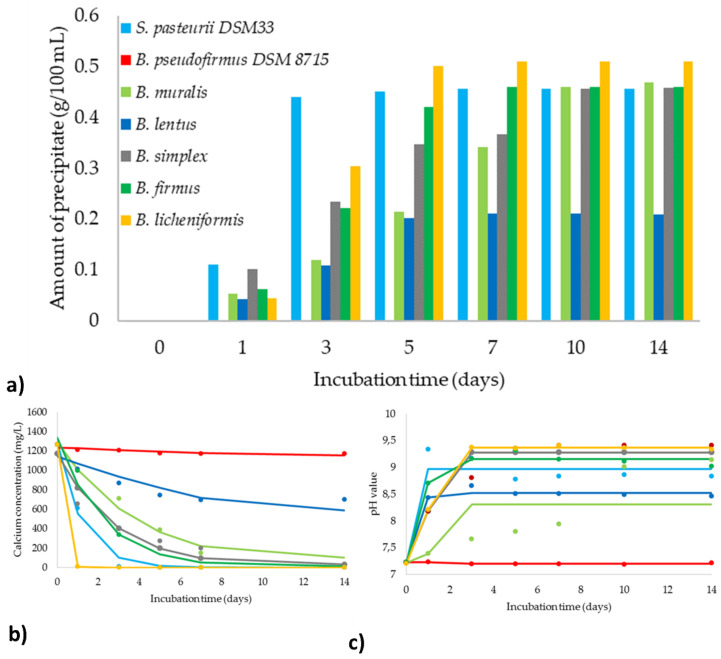
Evaluation of MICP process: (**a**) amount of precipitate; (**b**) pH monitoring; (**c**) calcium concentration (for (**b**,**c**) markers signify the experimental data; lines indicate predictive results).

Shortly after inoculation, within the first 6 h, the occurrence of deposits was noticed for altogether experiments except in the case of *B. muralis*, where the precipitate was observed within the first 24 h. Except for *B. lentus*, all other bacterial isolates produced a higher amount of precipitate than reference *S. pasteurii* DSM 33 at the initial phase. The only bacterial strain that stood out in inducing rate, as well as the amount of precipitate, was *B. licheniformis*. Comparing other isolates with ureolytic reference, it can be observed similarity in the amount of precipitate, but slower precipitate rate. A lower amount of precipitate was observed for *B. lentus*, which is in correlation with the gained results of ureolysis kinetics (Figure 4). All five ureolytic isolates were able to increase alkalinity through the MICP process for approximately 2 pH units (Figure 5b). The maximum pH values have been achieved after 3 days of incubation in the case of *B. lentus* and ureolytic reference, while the highest alkalinity for other isolates was observed within the first 5 days. In all experiments with ureolytic bacteria, calcium concentration decreased close to zero after approximately 5 h (Figure 5c). *B. licheniformis* completely accumulated free calcium from a liquid medium, decreasing the ion concentration by 99% in the first 24 h. On the other hand, calcium mineralization by *S. pasteurii* DSM 33 and *B. simplex* was slower, reducing the total concentration of calcium ions after a 3- and 5-day incubation period, respectively. In the case of *B. muralis* and *B. simplex*, instantaneous mineralization of calcium ions was not observed, and the concentration of free ions was lightly decreasing during incubation. It can be concluded that reduction kinetic for free calcium ions in accordance with the kinetic pathway of precipitation of calcium carbonate and pH changes during the MICP. Considering the fact that the negative charged bacterial walls act as absorbers for positive ions and mediate crystal formation as the center of nucleation, the number of viable and achievable bacterial cells did not determine in this experimental step. Furthermore, it can be noticed the impact of rapid and effective Urea hydrolysis in the first 72 h since the most intensive formation of precipitates was observed in this part of the incubation period. This is in accordance with the obtained results of ureolysis kinetics models. After this initial intensive phase in Urea hydrolysis as well as inorganic depositing, a stable phase during the MICP process with minimal changes in each sampling point was observed for each selected process parameter (amount of precipitate, pH monitoring, calcium concentration). The described behavior is similar for all tested bacteria, which can suggest that the kinetic of ureolysis has a great influence on the MICP process, regardless of the ureolytic bacterial strain. Table 1 also summarizes the regression coefficients of the observed mathematical models for MICP kinetics, which explain the trends of the investigated process. The fit between experimental measurements and model calculated results, as well as the residual analysis of the developed model in which the quality of the model fit was tested, are shown in Supplementary Table S3. The presented models are accurate according to the high coefficient of determination (greater than 0.846).

### 3.6. Precipitate Characterization

**Figure 6:**
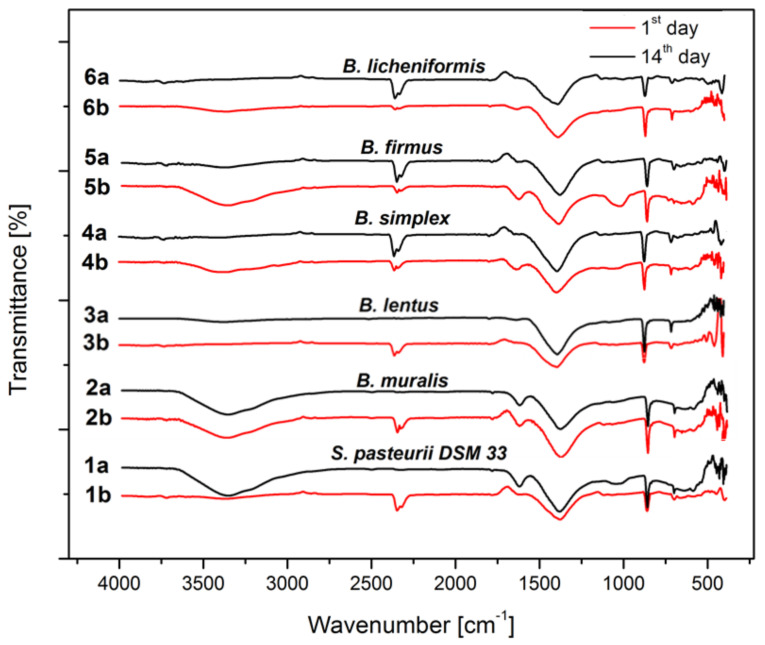
FTIR analysis (a—1st day; b—14th day; 1—*S. pasteurii* DSM 33; 2—*B. muralis*; 3—*B. lentus*; 4—*B. simplex*; 5—*B. firmus*; 6—*B. licheniformis*).

**Figure 7:**
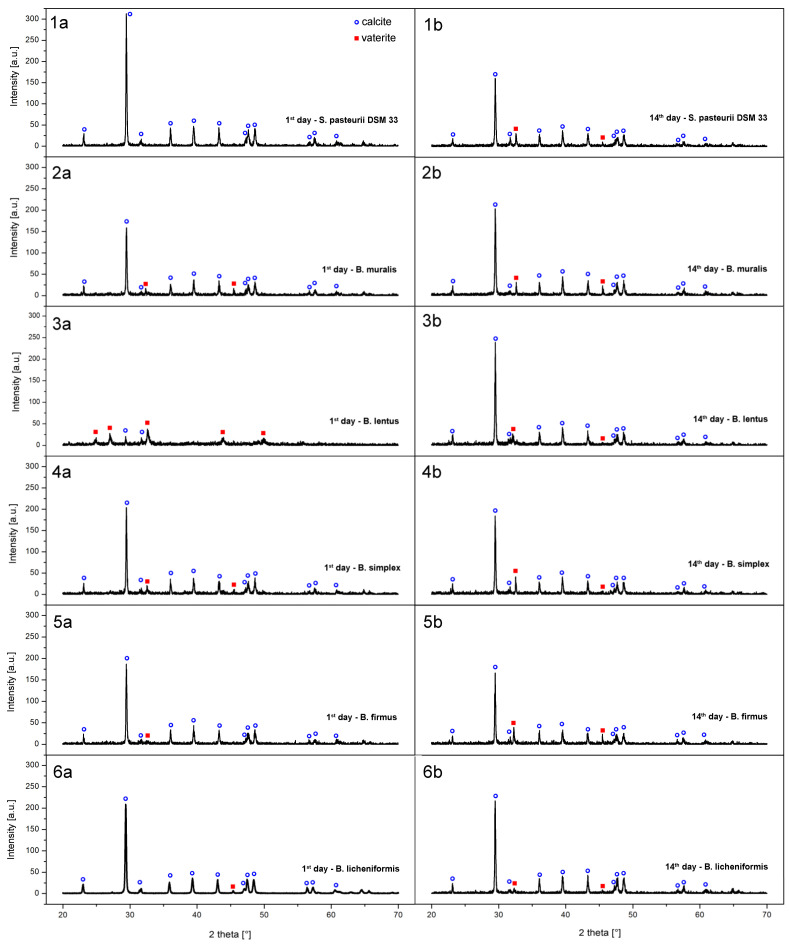
XRD spectra of CaCO_3_ precipitates (a—1st day; b—14th day; 1—*S. pasteurii* DSM 33; 2—*B. muralis*; 3—*B. lentus*; 4—*B. simplex*; 5—*B. firmus*; 6—*B. licheniformis*).

Considering that all tested ureolytic bacteria induce precipitation of inorganic deposits, the obtained precipitates (after the first and at the last incubation day) were subjected to structural characterization to observe the influence of incubation time on crystal formation, maturation, and morphology. The precipitates were first studied by using FTIR spectroscopy and X-ray diffraction (Figure 6 and Figure 7). According to the scientific data, the characteristic FTIR signals for CaCO_3_ are at ~1400 cm^−1^ (ν3—doubly degenerate planar asymmetric stretching), ~870 cm^−1^ (ν2—out-of-plane bending), and between 700 and 746 cm^−1^ (ν4—doubly degenerate planar bending). The absorption band 1100−1000 cm^−1^ can also be detected (ν1—symmetric stretching) but is less noticeable and does not have to be taken into consideration [48].

On the FTIR spectra of precipitates depicted in Figure 5, it can be observed from the first to the last incubation day the presence of carbonates because at least three characteristic peaks have appeared. For all overnight precipitates can be noticed the occurrence of peaks between 3650–3250, 1600, 1550−1480, and around 600 cm^−1^ which are amino-related and organic-related peaks. This suggests that the nutrient medium was not used completely after 24 h, while these peaks were less pronounced after the incubation period.

For XRD analysis (Figure 7), patterns are put on the same scale for better visual comparison, and diffraction peaks are identified with the reference patterns of calcite (COD ref. code 96−450−2444) and vaterite (COD ref. code 96–300–0001). It is evident from the XRD patterns for *B. muralis*, *B. firmus* and *B. simplex* that the calcite and vaterite phases are present from the first day, with much stronger well-shaped peaks of calcite form. Peak intensities of vaterite increase slightly after 14 days of incubation, indicating promoted crystallization orthorhombic phase. In these three samples, the vaterite content after 14 days is the highest in sample 5b, the precipitate from *B. firmus* incubation. The exception regarding phase formation dynamics may be found in the precipitate formed by *B. lentus* on the first day (sample 3a) of incubation. The identified phase was predominantly composed of vaterite. In contrast to this, the precipitate from *B. lentus* on the final day (sample 3b) was composed of a calcite phase mostly, with very low vaterite phase peak intensities. Finally, the precipitate from *B. licheniformis* showed a similar structure to the referent strain *S. pasteurii*, highly crystalline calcite phase on the first day, with traces of vaterite in precipitate from after 14 days. In short conclusion, the experimental conditions, in this case, show that all procedures produce mixed-phase powders after 14 days, predominantly made of calcite, while on the first day *B. lentus* form vaterite mostly, and *S. pasteurii* and *B. licheniformis* produce only calcite.

In brief reference to literature, the obtained results are in agreement with the report by Tepe et al. [49] in which is reported that *Bacillus* strains produce crystals with the mixture of calcite and vaterite. Depending on the experimental conditions, *S. pasteurii* DSM 33 showed precipitation of only calcite [50,51] or a mixture of calcite and vaterite [52]. Wei et al. [35] showed induction of calcite by *B. lentus*, while, different scientific groups reported that *B. licheniformis* can form only calcite pure precipitate [33,53]. In agreement with the obtained results also, a difference in XRD pattern during the incubation period of 14 days is observed by Ivanova et al. [54], where sediment isolate *B. licheniformis* induced vaterite single-phase structure formation at the beginning of the MICP process, while at the end of the observed period predominant calcite phase was detected.

According to Saracho et al. [55], CaCO_3_ morphology and polymorphism depend on the coupled effect of different rates of ureolytic activities and, consequently, precipitation kinetics, while the interaction between structural water and bacteria-specific amino acid groups on the cell walls is fundamental to stabilize morphology of crystals. Interestingly, the scientific relevant literature was reported calcite and aragonite as the most common bacterial carbonates [56], while this study observed only calcite and vaterite in all tested samples. This can suggest that precipitation of vaterite might be more common than expected, or this phase is subjected to rapid calcite changes during the maturation of the crystals.

**Figure 8:**
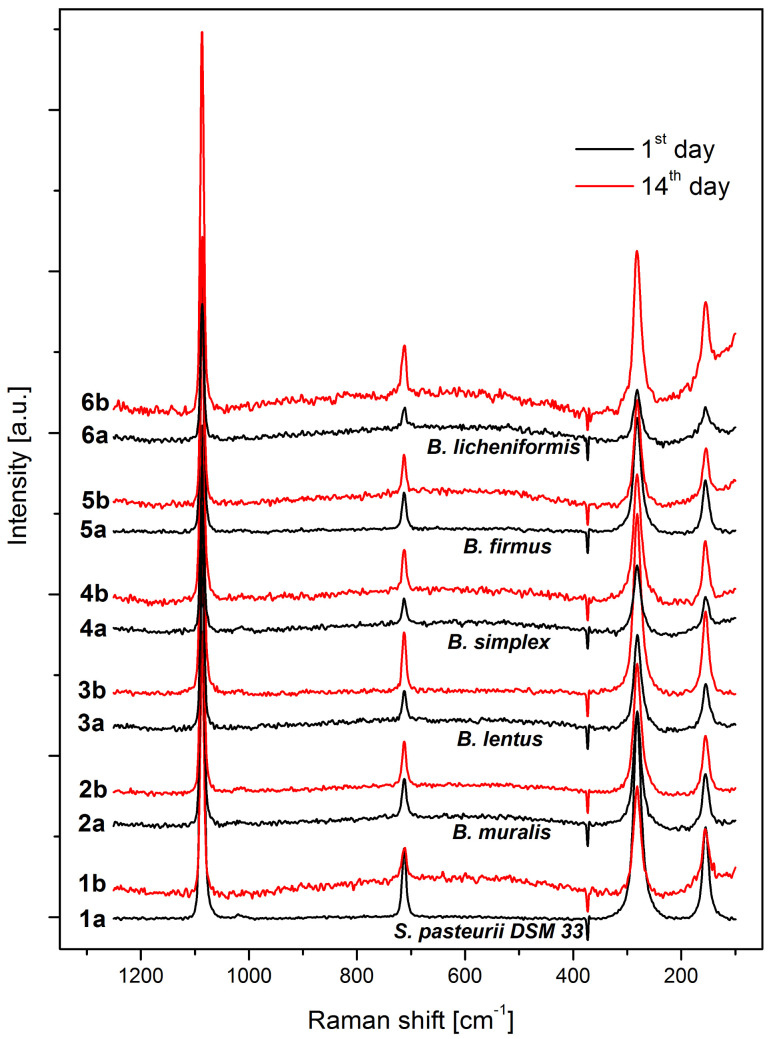
RAMAN spectra CaCO_3_ precipitates (a—1st day; b—14th day; 1—*S. pasteurii* DSM 33; 2—*B. muralis*; 3—*B. lentus*; 4—*B. simplex*; 5—*B. firmus*; 6—*B. licheniformis*).

To investigate more thoroughly the obtained precipitates and more closely study potential differences, all samples were analyzed by Raman spectroscopy. The results show that calcite bands are dominantly present in general (Figure 8), given that the excitation at 1086 cm^−1^ was detected as the high-intensive peak for all samples. In the same position two close peaks of vaterite, at 1080 cm^−1^ and 1090 cm^−1^ [57], are probably in superposition with the calcite, and cannot be distinguished. The Raman spectra also showed Raman modes of the hexagonal calcite crystal lattice at 284 cm^−1^ and 157 cm^−1^. Calcite is additionally confirmed by the peak at 712 cm^−1^, while the characteristic peak for vaterite was not clearly observed. It may be assumed that broadening of calcite peak at 284 cm^−1^ in its base is caused by vaterite modes found at 299 cm^−1^ and 365 cm^−1^, slightly more noticeable in precipitates collected after 14 days [57].

**Figure 9:**
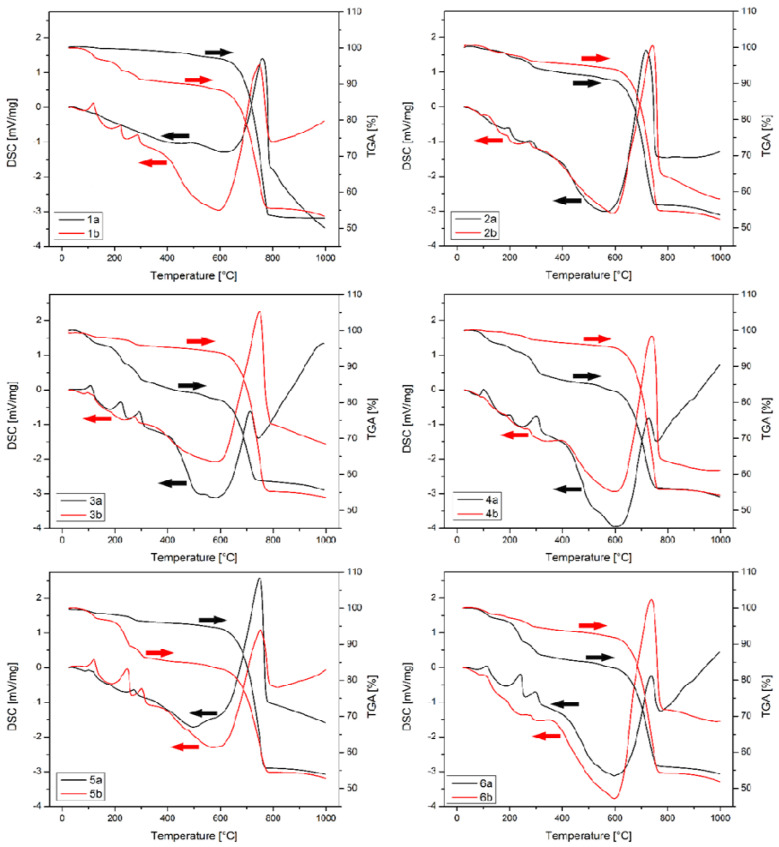
DTA/TGA of CaCO_3_ precipitates (a—1st day; b—14th day; 1—*S. pasteurii* DSM 33; 2—*B. muralis*; 3—*B. lentus*; 4—*B. simplex*; 5—*B. firmus*; 6—*B. licheniformis*; black arrow—1st day; red arrow—14th day).

According to the structure characterization above, DTA/TGA analysis had shown consistent results (Figure 9). Around 20 mg of each sample was heated to 1000 °C, at a rate of 10 °C/min. Two stages in the thermal analysis may be noticed, below and above 400 °C. Below 400 °C three peaks are noticeable followed by a minor weight loss. The first one occurring around 115 °C is assigned to evaporation of humidity. The other two peaks around 220 and 290 °C probably represent the decomposition of organic residues from the incubation procedure or trapped bacterial spores or cells that previously were nucleation centers, with confirmed presence in all samples with FTIR analysis. Weight loss dynamics below 400 °C vary to a certain extent probably due to a difference in precipitate microstructure (density and particle size), as well as absorbent water. The second part of the DTA diagram shows rapid weight loss above 600 °C, followed by a strong peak in the DSC signal due to carbonates decomposition.

**Figure 10:**
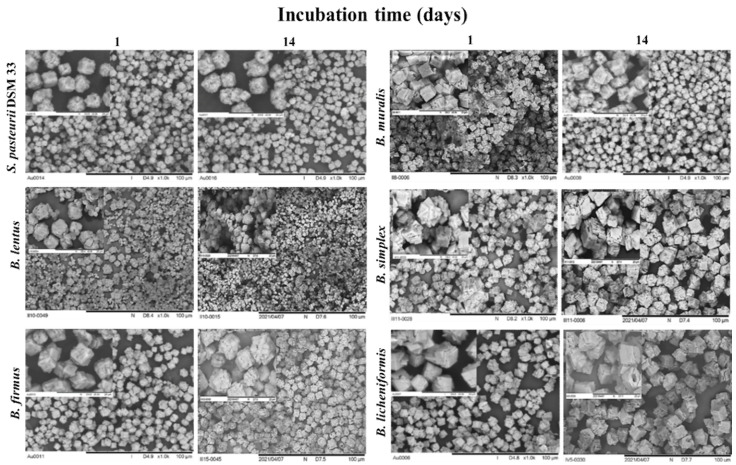
Low magnification SEM images (1000×) and their corresponding higher magnification as an inset in the left upper corner (5000×) for the obtained precipitates.

SEM analysis confirmed XRD and Raman results regarding crystallinity in all samples. The morphology and crystallite size of CaCO_3_ crystals induced by the bacterial activity depends mainly on the relative crystal growth rate in all directions. Moreover, the growth rate depends on the cell surface processes and is highly sensitive to the effects of environmental conditions [58]. SEM micrographs (Figure 10) confirm a high degree of crystallinity on the first incubation day, in most samples, which is defined by well-developed crystal forms characteristic for CaCO_3_ [59], with average sizes in a range of micrometers. Precipitates from the last day are all characterized by a high degree of crystallinity. After the first day of the incubation, CaCO_3_ crystals induced by *S. pasteurii* cells are represented by rhombohedral morphology of calcite, with a similar pattern at the end of the incubation period. After the first day incubation period, the precipitate, which forms in the presence of *B. muralis* cells, manifests the topography of spherical aggregates of calcite, while edges and corners become less sharp during the time. On the other hand, crystals induced by *B. lentus* cells have evident different particle size distribution since the size of the obtained forms varies the most for both samples during the incubation period. The morphology of CaCO_3_ crystals during the incubation of *B. simplex* undergoes changes from the flower-like calcite to more rhombohedral shapes with constant particle size distribution. Similar spherical aggregates were observed for *B. firmus* during the MICP process. The regular rhombohedral morphology was observed for crystals induced by *B. licheniformis*, where the crystals are visibly grown from square layer by layer from the beginning of the incubation period.

The particle size distribution was narrow for referent strain *S. pasteurii* DSM 33 precipitates, with sizes ranging from 3 to 5 μm, confirming the phase characterization above. In the case of *B. muralis*, *B. simplex*, and *B. firmus* precipitates, SEM micrographs show similar morphology and particle shape and size, which is also in line with XRD patterns. *B. lentus* seems to promote a rather bimodal particle distribution, especially on the first day of incubation. Judging from the XRD pattern, lower crystallinity was found for *B. lentus* during the first day (Figure 7, sample 3a), which implies that this bacterium promotes relatively slower crystallization of both phases (calcite and vaterite) in given conditions, compared to other tested strains. Vaterite phase later evolved to calcite, showing increased particle sizes on micrographs (Figure 7, sample 3b) and better crystallinity on XRD. *B. licheniformis* had shown a rather pure calcite phase and well-developed square-shaped particles on the first day of incubation, comparable to the referent strain *S. pasteurii* DSM 33. The precipitate from the 14th day of incubation (Figure 7, sample 6b) showed even bigger particles than the reference strain, which supports the biggest yield of synthesized powder among all tested bacteria.

## 4. Conclusions

The microbiologically induced CaCO_3_ precipitation may be conducted through comprehensive profiling of bacterial strains with appropriate ureolytic and MICP kinetics, while characterization of precipitate induced by chosen bacteria may be an advanced approach to understanding ecological and physiological responses at the bacterial strain level during the MICP process. In brief, this study presented that different ureolytic *Bacillus* strains isolated from alkaline soils not only adapt to induce the MICP during incubation in an adequate nutrient medium but that this process is very sensitive and specific to the bacterial strain level. Consequently, the sensitivity of the MICP process on the observed level may implicate examination of conditions near those of the real environment of application of the engineered MICP process. However, using an effective *Bacillus* strain such as *Bacillus licheniformis*, that can induce the desired amount and characteristics of CaCO_3_ precipitate in variable environmental conditions, offers potential for high-efficiency processes in different fields such as civil engineering, bioremediation, and biotechnological applications, geotechnical and ecological engineering, bio-based solution for soil, sand, cement-based matrix, etc.

## Figures and Tables

**Table 1:** Regression coefficients.

**Kinetics of ureolysis (** Section 3.4 **)**								
**Parameters**	**Coefficient**	**Referent strain**		**Natural isolates**				
		*S. pasteurii*	*B. pseudofirmus*	*B. muralis*	*B. lentus*	*B. simplex*	*B. firmus*	*B. licheniformis*
**Urea** **concentration**	**k_Urea_**	0.10	0.00	0.02	0.01	0.03	0.03	0.04
	**c_Urea_**	19.45	19.878	19.95	19.42	19.55	19.99	19.99
**pH** **value**	**d**	9.15	7.24	8.87	8.14	9.2	9.14	8.812
	**a**	7.19	7.19	7.27	7.36	7.45	7.19	7.2
	**c**	3.07	5.54	8.13	13.85	15.35	12.33	11.40
	**b**	1.37	5.28	7.43	2.99	4.01	2.37	15.30
**Cell** **concentration**	**d**	9.90	6.68	6.567	8.11	8.18	8.6	11.66
	**a**	5.55	5.05	5.61	6.3	5.75	6.2	5.03
	**c**	30.00	20.89	18.71	30.00	9.16	15.20	32.15
	**b**	0.79	1.96	4.49	1.78	4.93	2.95	0.8
Kinetic of the MICP process (Section 3.5)								
**Parameters**	**Coefficient**	*S. pasteurii*	*B. pseudofirmus*	*B. muralis*	*B. lentus*	*B. simplex*	*B. firmus*	*B. licheniformis*
**Amount of** **precipitate**	**d**	0.46	0.001	0.57	0.21	0.56	0.47	0.51
	**a**	0.001	0.001	0.001	0.001	0.001	0.001	0.001
	**c**	1.32	5.78	5.88	2.98	3.69	3.04	2.82
	**b**	4.09	2.56	2.06	5.97	1.22	3.75	6.00
**pH value**	**d**	8.97	7.20	8.31	8.53	9.28	9.15	9.37
	**a**	7.23	7.23	7.22	7.23	7.23	7.23	7.21
	**c**	0.50	1.78	1.07	0.89	1.01	0.95	1.01
	**b**	19.17	19.06	24.99	22.89	19.00	25.00	24.55
**Calcium concentration**	**k_precipitate_**	0.84	0.01	0.251	0.07	0.36	0.45	4.84
	**c_Ca_^2+^**	1282.34	1237.18	1294.89	1146.27	1171.96	1340.89	1266.00

## Data Availability

The data presented in this study are available in cited references and on request from the corresponding authors.

## Supplementary Materials

The supplementary materials are available online at https://www.mdpi.com/article/10.3390/microorganisms9081691/s1. Table S1: Standard Score Analyses Database, Table S2: The “goodness of fit” tests for Urea concentration, pH value and cell concentration prediction models, Table S3: The “goodness of fit” tests for amount of precipitate, pH value and reduction of Ca^2+^ ions prediction models.
