# Supplementary material for: Comprehensive Profiling of Microbiologically Induced CaCO3 Precipitation by Ureolytic Bacillus Isolates from Alkaline Soils
Source: Microorganisms. 2021 Aug 9;9(8):1691. doi: 10.3390/microorganisms9081691 (PMC8400936; doi:10.3390/microorganisms9081691)
Supplement: Supplementary file 1 [file microorganisms-09-01691-s001.zip › microorganisms-1292147-supplementary.pdf]

Supplementary Table S1. Standard Score Analyses Database

Parameters	Spore formation	Optimal pH growth value	Urea agar		Urea broth			$CaCO_3$ formation on solid nutrient media		$CaCO_3$ formation in liquid nutrient media		Standard score
			growth	color change	turbidity	color change	$CaCl_2$ addition	Urea-$CaCl_2$	EK-$CaCl_2$	Urea-$CaCl_2$	EK-$CaCl_2$	
Min	0	7	0	0	0	0	0	0	0	0	0	
Max	1	11	1	1	2	3	3	1	1	1	1	**SUM**
**Weights**	**0.05**	**0.45**	**0.001**	**0.014**	**0.001**	**0.014**	**0.02**	**0.075**	**0.075**	**0.15**	**0.15**	**1**
Polarity	+	9	+	+	+	+	+	+	+	+	+	
**Bacteria**												
S. pasteurii	1	1	1	1	1	1	1	1	0	1	0	**0.77**
B. pseudofirmus	1	1	0	0	0.5	0	0	0	1	0	1	**0.73**
**I**$_1$	1	0	1	1	0	0	0	1	1	0	1	0.36
**I**$_2$	1	1	1	0	0	0	0	0	0	0	0	0.50
**I**$_3$	1	1	1	1	0	0	0	1	1	0	0	0.66
**II**$_1$	1	0	1	1	0.5	0	0	1	1	1	0	0.37
**II**$_2$	1	0	1	1	0	0	0	1	1	1	0	0.36
**II**$_3$	1	1	1	1	0.5	0	0	0	0	0	0	0.52
**II**$_4$	1	1	1	1	0	0	0	1	1	1	0	0.81
**II**$_5$	1	1	1	1	0.5	0	0	0	1	1	0	0.74
**II**$_6$	1	0	1	1	0.5	0	0	0	1	1	0	0.29
**II**$_7$	1	1	1	1	0.5	0	0	1	1	0	1	0.82
**II**$_8$	1	1	1	1	1	0	0	1	1	1	1	**0.966**
**II**$_9$	1	1	1	1	0.5	0	0	1	0	1	1	0.891
**II**$_{10}$	1	1	1	1	0.5	0	0	1	1	1	1	**0.97**
**III**$_1$	1	0	1	1	0.5	0	0	1	1	1	1	0.52
**III**$_2$	1	1	1	1	0.5	0	0	0	1	0	1	0.74
**III**$_3$	1	1	1	0	0.5	0	0	0	0	0	1	0.65
**III**$_4$	1	1	1	0	0.5	0	0	0	1	1	1	0.88
**III**$_5$	1	1	1	0	0.5	0	0	1	1	0	1	0.80
**III**$_6$	1	1	1	1	0.5	0	0	1	1	0	1	0.82
**III**$_7$	1	1	1	1	0.5	0	0	1	1	0	1	0.82
**III**$_8$	1	1	1	1	0.5	0	0	1	0	0	1	0.74
**III**$_9$	1	1	1	1	0.5	0	0	1	1	0	1	0.82
**III**$_{10}$	1	1	1	1	0.5	0	0	1	1	0	1	0.82
**III**$_{11}$	1	1	1	1	0.5	0	0	1	1	1	1	**0.97**
**III**$_{12}$	1	1	1	1	0.5	0	0	0	1	0	1	0.74
**III**$_{13}$	1	0	1	0	0.5	0	0	0	0	0	1	0.20
**III**$_{14}$	1	1	1	0	0.5	0	0	0	1	1	1	0.88
**III**$_{15}$	1	1	1	1	0.5	0	0	1	1	1	1	**0.97**
**III**$_{16}$	1	1	1	1	0.5	0.33	0	1	1	0	1	0.82
**III**$_{17}$	1	0	1	1	0.5	0	0	1	1	0	1	0.37
**III**$_{18}$	1	0	1	1	0.5	0	0	1	1	0	1	0.37
**III**$_{19}$	1	0	1	1	0.5	0	0	1	1	1	1	0.52
**III**$_{20}$	0	0	1	1	0.5	0.33	0.33	1	0	1	1	0.40
**IV**$_1$	1	0	1	1	0.5	0	0	0	0	1	1	0.37
**IV**$_2$	1	1	1	1	0.5	0	0	0	1	0	0	0.59
**IV**$_3$	1	0	1	1	0.5	0	0	0	1	0	1	0.29
**IV**$_4$	1	0	1	1	0.5	0	0	1	1	0	1	0.37
**IV**$_5$	1	1	1	1	0.5	0	0	1	1	1	1	**0.97**
**IV**$_6$	1	1	1	1	0.5	0	0	0	1	0	0	0.59
**IV**$_7$	0	1	1	1	0.5	0.33	0	0	1	0	0	0.54
**IV**$_8$	0	0	1	1	0.5	0.33	0	0	1	0	1	0.24

SUM 1.0

**IV_9**	0	0	1	0	0.5	0.08	0	0	1	1	1	0.38
**IV_{10}**	1	1	1	1	0.5	0.33	0.17	1	1	1	0	0.82

Supplementary Table S2. The 'goodness of fit' tests for Urea concentration, pH value and cell concentration prediction models

	χ^2	RMSE	MBE	MPE	r^2	Skew	Kurt	Mean	SD	Var.
**Urea concentration**										
S. pasteurii	0.219	0.362	0.098	28.090	0.998	-0.368	-1.110	0.098	0.368	0.135
B. pseudofirmus	0.003	0.045	-0.023	0.168	0.876	-0.243	-1.307	-0.023	0.040	0.002
B. muralis	2.282	1.170	0.056	8.739	0.955	-0.611	-0.244	0.056	1.232	1.518
B. lentus	1.308	0.886	0.174	4.396	0.944	-0.615	1.238	0.174	0.916	0.839
B. simplex	2.840	1.305	-0.067	15.697	0.963	0.783	1.236	-0.067	1.374	1.888
B. firmus	5.181	1.763	0.205	38.324	0.964	-0.048	-1.122	0.205	1.846	3.407
B. licheniformis	0.765	0.677	0.099	9.751	0.991	-0.415	1.275	0.099	0.706	0.499
**pH value**										
S. pasteurii	0.015	0.098	-0.016	0.970	0.978	0.207	-0.620	-0.016	0.101	0.010
B. pseudofirmus	0.001	0.024	0.004	0.229	0.821	0.408	2.083	0.004	0.025	0.001
B. muralis	0.022	0.119	0.024	1.076	0.974	0.540	0.177	0.024	0.122	0.015
B. lentus	0.017	0.103	0.021	1.058	0.929	0.341	0.500	0.021	0.106	0.011
B. simplex	0.022	0.120	0.010	1.316	0.978	-0.975	-0.137	0.010	0.125	0.016
B. firmus	0.014	0.093	-0.001	0.835	0.986	0.162	0.776	-0.001	0.097	0.009
B. licheniformis	0.019	0.110	0.011	1.113	0.980	-0.102	-1.110	0.011	0.115	0.013
**Cell concentration**										
S. pasteurii	0.158	0.317	0.085	2.303	0.935	1.956	5.245	0.085	0.321	0.103
B. pseudofirmus	0.019	0.109	-0.003	1.572	0.966	-1.364	1.589	-0.003	0.115	0.013
B. muralis	0.005	0.056	0.005	0.607	0.985	0.535	1.550	0.005	0.058	0.003
B. lentus	0.067	0.206	-0.001	2.491	0.883	-1.190	1.129	-0.001	0.216	0.047
B. simplex	0.062	0.199	0.013	2.606	0.964	0.132	-1.816	0.013	0.208	0.043
B. firmus	0.039	0.157	0.012	1.897	0.977	-0.551	-0.704	0.012	0.164	0.027
B. licheniformis	0.042	0.164	0.034	1.731	0.990	0.746	0.091	0.034	0.168	0.028

χ^2– reduced chi-square; RMSE – root mean square error; MBE – mean bias error; MPE – mean percentage error; r^2 - coefficient of determination; Skew. – skewedness; Kurt. – kurtosis; SD – standard deviation; Var. – variance.

Supplementary Table S3. The 'goodness of fit' tests for amount of precipitate, pH value and reduction of Ca^{2+} ions prediction models

	χ^2	RMSE	MBE	MPE	r^2	Skew	Kurt	Mean	SD	Var.
**Mass of precipitate**										
S. pasteurii	0.000	0.001	0.000	0.388	1.000	-2.187	5.195	0.000	0.001	0.000
B. pseudofirmus	0.000	0.000	0.000	0.000	1.000	0.000	0.000	0.000	0.000	0.000
B. muralis	0.001	0.023	0.005	14.280	0.986	0.255	-1.065	0.005	0.024	0.001
B. lentus	0.001	0.016	0.006	14.039	0.980	2.638	6.968	0.006	0.016	0.000
B. simplex	0.000	0.014	0.001	4.022	0.993	0.321	-1.369	0.001	0.015	0.000
B. firmus	0.001	0.022	0.007	14.109	0.991	1.939	4.086	0.007	0.022	0.000
B. licheniformis	0.001	0.017	0.006	13.954	0.997	2.526	6.478	0.006	0.017	0.000
**pH value**										
S. pasteurii	0.086	0.192	0.000	1.790	0.909	1.166	0.130	0.000	0.208	0.043
B. pseudofirmus	0.000	0.009	0.000	0.091	0.846	1.147	2.196	0.000	0.009	0.000
B. muralis	0.019	0.090	-0.050	0.614	0.975	-1.723	2.205	-0.050	0.081	0.006
B. lentus	0.008	0.059	0.000	0.447	0.983	1.889	4.511	0.000	0.063	0.004
B. simplex	0.093	0.199	0.000	1.476	0.935	-2.263	5.403	0.000	0.215	0.046
B. firmus	0.016	0.082	0.000	0.536	0.985	0.723	2.936	0.000	0.088	0.008
B. licheniformis	0.001	0.020	0.000	0.140	0.999	0.517	1.989	0.000	0.021	0.000
**Reduction of calcium ions**										
S. pasteurii	6216	45.519	-12.799	166850	0.992	-0.521	2.257	-12.799	47.851	2289.754
B. pseudofirmus	920	17.509	0.005	1.297	0.728	0.811	-1.149	0.005	19.180	367.859
B. muralis	13964	68.225	-14.483	4372163	0.980	0.564	0.328	-14.483	73.033	5333.842
B. lentus	20593	82.851	1.667	8.703	0.836	0.810	-1.931	1.667	90.740	8233.722
B. simplex	26567	94.105	12.727	1402613	0.949	-1.113	0.705	12.727	102.14	10432.49
B. firmus	24784	90.891	-23.678	3173533	0.973	1.070	2.027	-23.678	96.128	9240.688
B. licheniformis	3.123	1.020	0.417	66.662	1.000	2.449	6.000	0.417	1.020	1.041

χ^2– reduced chi-square; RMSE – root mean square error; MBE – mean bias error; MPE – mean percentage error; r^2 - coefficient of determination; Skew. – skeweness; Kurt. – kurtosis; SD – standard deviation; Var. – variance.
